# Sir Henry Burdett

**Published:** 1920-05-08

**Authors:** 


### The Hospital

The Workers' Newspaper of Administrative Medicine and Institutional Life, Administration, National Insurance and Health.

No. 1770, Vol. LXVIII. SATURDAY, MAY 8, 1920. PRICE TWOPENCE

### SIR HENRY BURDETT.

As was announced in the later edition of The Hospital last week, Sir Henry Charles Burdett passed peacefully away on Thursday evening, April 29, at his home in Porchester Terrace in the seventy-fourth year of his age. He had been seriously ill since February, but early in April a marked improvement in his condition led to a hope that possibly his naturally fine constitution would triumph. The improvement turned out to be but temporary, and the hope was never realised. Founder of The Hospital in 1886, and sole Editor from that time to the present, the loss of him we mourn to-day is inevitably profound and far-reaching; to a degree rare even amongst founder-editors he was the moving spirit of the newspaper which he created. Right up to the onset of his illness he had been contributing a series of fervent leading articles, in which his personality was easily to be recognised, embodying and epitomising his gospel of life and of duty in relation to institutional affairs. He had been one of the first to appreciate the post-war situation as it affects voluntary institutions; and he foresaw months ago what we can all see to-day, the urgency of the reconstruction of hospital finance. More than that, he spoke out plainly, repeatedly, even vehemently; and if his warnings were here and there neglected, at least it can be said that in other places they are bearing fruit.

In the Memoir which will be found on a later page, Sir Henry Burdett's career and personality are envisaged from diverse points of view. The qualities there revealed he brought also to the work of an editor. Unsparing of himself, he exacted an equally high standard of endeavour from those who worked under him; and if he had small tolerance for a shirker, he was fully appreciative of all who would render him loyal service to the best of their particular abilities. Not afraid to strike hard when he felt it to be necessary, he was yet unwilling to wound anyone if he knew a way to avoid it. Ardent in controversy, and ever ready to accept a challenge, he yet bore no malice and was free from personal grudge against opponents. One of the oldest editors in London, he was yet open-minded concerning any modern journalistic teachings worthy of an honourable trial. Thoroughly aware of the importance of type and editorial presentation in a newspaper, he knew that neither can long disguise bad or insincere writing. Perhaps he was seen at his best in the two remarkable series of Reports on hospitals of the United Kingdom which he wrote, one between 1891 and 1895, and the other between 1909 and 1914, for this journal. These special reports are probably unique in London journalism, in that the Editor was his own special correspondent: he went himself all over the kingdom to inspect the institutions which he described, and his reports are models of fairness and thoroughness. Whenever he felt it necessary to criticise, no one ever suggested that his criticism was not honest; and it was seldom indeed that its validity and authority were disputed. Small institutions were not neglected, though due proportion was observed in the space allotted to them as compared with the larger establishments. It was no small tribute to the sagacity of this voluntary inspector of voluntary institutions, as well as to the general confidence in which his judgment on hospital matters was held, that all doors were thrown freely open to him whenever and wherever he sought entrance.

In the hospital world both of Europe and of America it is perhaps a truism to say that he was an institution: the acknowledged doyen of workers still upon the active list. Expressions of sorrow and sympathy have reached us from every circle. There is no civilised country without a hospital, and there is no hospital, whether voluntary or State-supported, in which the death of Sir Henry Burdett, in its degree, will not be felt. His activities extended through a public life of crowded years, and were so diverse as to be almost bewildering. Nearly all of them were connected with hospitals, or organisations ancillary thereto. His energy was incessant, and would have worn out most men's bodies by middle life. One of his biographers in the daily press calls him a "human dynamo," and Mr. T. P. O'Connor says that he once wondered whether he was a "man or a machine." What is the answer? He was a man; and if there seemed something super-human about his expenditure of energy, it is due to the fact that he was a man inspired by one idea, by an ideal. The central article of his faith was also the central fact of his experience: he lived up to his ideal. This faith was contained in a phrase which he was never tired of using, the gospel of "personal service to the sick in the days of health." To Sir Henry Burdett these words never became hackneyed, stale, or meaningless. If he used them in season and out of season, it was because he was unable to find a better or simpler formula for his belief, nor one which more completely expressed his attitude towards life. Another of his favourite expressions was that "every man is the trustee of his own life"; by which he meant that each one of us who is born comes into the world pre-destined to be a steward; that life is a stewardship; and that not even Death, perhaps, relieves one of the trust. When the news of the death of Florence Nightingale was conveyed to him, he said quietly: "Her trusteeship is over. . . But the trust continues. The work goes on."

